# Safety assessment of inhaled xylitol in mice and healthy volunteers

**DOI:** 10.1186/1465-9921-5-13

**Published:** 2004-09-16

**Authors:** Lakshmi Durairaj, Janice Launspach, Janet L Watt, Thomas R Businga, Joel N Kline, Peter S Thorne, Joseph Zabner

**Affiliations:** 1 Department of Medicine, Roy J. and Lucille A. Carver College of Medicine, University of Iowa, Iowa City, Iowa, USA; 2 Department of Occupational and Environmental Health, College of Public Health University of Iowa, Iowa City, Iowa, USA

## Abstract

**Background:**

Xylitol is a 5-carbon sugar that can lower the airway surface salt concentration, thus enhancing innate immunity. We tested the safety and tolerability of aerosolized iso-osmotic xylitol in mice and human volunteers.

**Methods:**

This was a prospective cohort study of C57Bl/6 mice in an animal laboratory and healthy human volunteers at the clinical research center of a university hospital. Mice underwent a baseline methacholine challenge, exposure to either aerosolized saline or xylitol (5% solution) for 150 minutes and then a follow-up methacholine challenge. The saline and xylitol exposures were repeated after eosinophilic airway inflammation was induced by sensitization and inhalational challenge to ovalbumin. Normal human volunteers underwent exposures to aerosolized saline (10 ml) and xylitol, with spirometry performed at baseline and after inhalation of 1, 5, and 10 ml. Serum osmolarity and electrolytes were measured at baseline and after the last exposure. A respiratory symptom questionnaire was administered at baseline, after the last exposure, and five days after exposure. In another group of normal volunteers, bronchoalveolar lavage (BAL) was done 20 minutes and 3 hours after aerosolized xylitol exposure for levels of inflammatory markers.

**Results:**

In naïve mice, methacholine responsiveness was unchanged after exposures to xylitol compared to inhaled saline (p = 0.49). There was no significant increase in Penh in antigen-challenged mice after xylitol exposure (p = 0.38). There was no change in airway cellular response after xylitol exposure in naïve and antigen-challenged mice. In normal volunteers, there was no change in FEV1 after xylitol exposures compared with baseline as well as normal saline exposure (p = 0.19). Safety laboratory values were also unchanged. The only adverse effect reported was stuffy nose by half of the subjects during the 10 ml xylitol exposure, which promptly resolved after exposure completion. BAL cytokine levels were below the detection limits after xylitol exposure in normal volunteers.

**Conclusions:**

Inhalation of aerosolized iso-osmotic xylitol was well-tolerated by naïve and atopic mice, and by healthy human volunteers.

## Background

Human airway surface is covered by a thin layer of liquid (airway surface liquid [ASL]) that contains many antimicrobial substances including lysozyme, lactoferrin, human β defensins, and the cathelicidin LL-37 [1-4]. The antibacterial activity of most of these innate immune mediators is salt-sensitive; an increase in salt concentration inhibits their activity [5]. An equally interesting feature of these antimicrobial factors is that their activity is increased by low ionic strength [6-9]. Lowering the ASL salt concentration might therefore increase the efficacy of the innate immune system and thereby decrease or prevent airway infections.

The airway epithelium is water-permeable [10]. When large volumes of ionic, isotonic liquid are placed on the apical surface, active salt and liquid absorption occurs [11,12]. If water were added to the airway surface, the salt concentration would quickly return to starting values. Thus, lowering of ASL salt concentration is best accomplished using a nonionic osmolyte with low transepithelial permeability. The osmolyte should not provide a ready carbon source for bacteria, and should be safe in humans. One such promising osmolyte is xylitol, a five-carbon sugar that has low transepithelial permeability, is poorly metabolized by bacteria and can lower the salt concentration of both cystic fibrosis (CF) and non-CF epithelia *in vitro *[13]. Xylitol is an artificial sweetener that has been successfully used in chewing gums to prevent dental caries [14,15]; it has been used as an oral sugar substitute without significant adverse effects [16]. It has also been used in lozenges and syrup and has been shown to decrease the incidence of acute otitis media by 20–40% [17]; nasal application to normal human subjects was found to decrease colonization with coagulase negative staphylococcus [13]. There are no studies, to our knowledge, examining the effects of inhalation of aerosolized xylitol by experimental animals or humans.

Osmotic agents such as hypertonic saline, which is ionic, and nonionic mannitol, dextran and lactose, have been used in human subjects to increase mucus clearance [18-23]. However, some of these agents can serve as a carbon source for bacteria and can cause bronchospasm due to the tonicity. Nebulization of distilled water has been shown to increase airway resistance significantly in asthmatic subjects leading to subsequent use as a bronchoprovocative agent [24-26]. Both hypotonic and hypertonic saline solutions can provoke bronchospasm (a 20% drop in Forced Expiratory Volume in 1 second, FEV1) in asthmatic subjects but not in normal volunteers [26]. Furthermore, inhalation of 20% dextrose in the same study produced bronchospasm similar to exposure to water or hypertonic saline raising the possibility that osmolarity of the solution is the important determinant of bronchial reactivity.

In subjects with bronchiectasis, inhalation of dry powdered mannitol can increase the clearance of mucus without affecting lung function [27]. However, in a different study on subjects with CF, inhaled mannitol caused a small but significant decline in FEV1 (7.3%, P = 0.004) from baseline immediately after inhalation, which returned to baseline by the end of the study [28].

We hypothesized that aerosolized iso-osmolar xylitol is safe and well-tolerated well by normal subjects. We compared the safety and tolerability of aerosolized xylitol with normal saline, and carried out additional exposure studies using mice.

## Methods

### Safety in normal mice

All experiments were reviewed and approved by the animal care and use committee of the University of Iowa. Except during exposures and evaluation, mice were allowed access to food and water ad libitum. C57bl/6 mice (Jackson Lab, Bar Harbor, MA) underwent baseline methacholine challenge test using a whole-body plethysmograph (Buxco Electronics, Troy, NY) as previously described [29]. Respiratory pattern changes were expressed as enhanced respiratory pause (Penh), which correlates with changes in airway resistance. Airway resistance was expressed as follows: P_enh _= ([T_e _/0.3 T_r _] - 1) × [2P_ef_/3P_if _], where *P*_enh _equals enhanced pause, *T*_e _equals expiratory time (in seconds), *T*_r _equals relaxation time (in seconds), *P*_ef _equals peak expiratory flow (in milliliters per second), and *P*_if _equals peak inspiratory flow (in milliliters per second).

Mice (6/group) were exposed to aerosolized saline (0.9 % NaCl) or aerosolized xylitol (5% solution in water, equimolar to the NaCl) for 150 minutes in an exposure chamber; all mice were evaluated for bronchial hyperreactivity to inhaled methacholine (using the Buxco whole body plethysmography system) before and after the exposures; other mice were monitored periodically during exposure by whole body plethysmography. All mice underwent whole lung lavage the next day for cell count and differential. After euthanasia, the trachea was cannulated, and the lungs were lavaged with 3.0 mL of sterile normal saline (0.9% NaCl). The lavage samples were immediately processed for total and differential (with Diff Quick Stain; Baxter Scientific, Miami, FL) cell counts. In a separate group of naïve mice, whole body plethysmography was used to monitor Penh, respiratory rate, and tidal volume periodically during exposure to xylitol and saline for 10, 20, 40, and 80 minutes for a cumulative total dose of 150 minutes.

### Safety in hypersensitive mice

We repeated the saline and xylitol exposure protocol to 2 more groups of six mice each after they were sensitized to and challenged with an antigen [30]. Mice were sensitized to OVA (10 μg with 1 mg alum, i.p.) on days 0 and 7, then challenged with aerosolized OVA (1% solution, 30 minutes) on days 14 and 16. Filtered air was passed at 6 L/min through an Aero-Tech nebulizer (CIS-US Inc) to generate an aerosol. The size distribution of the aerosol was determined using a particle counter (Aerodynamic Particle Sizer, TSI Incorporated). The aerosol sizes were distributed log normally with a count median aerodynamic diameter of 0.82 microns and geometric standard deviation (GSD) of 1.46 microns. A mean OVA concentration of 3.8 ng/ml was measured in the chamber during the exposures. The mice underwent a baseline methacholine challenge on day 17 and subsequently underwent exposures to saline and xylitol using the same protocol described for the naïve mice. Three mice per group underwent whole lung lavage 24 hours after exposure for cell count and differential.

Given the concerns that have been raised about the reliability of airway resistance measurement by Buxco equipment, in a select number of mice we confirmed airway hyperresponsiveness using invasive measurement. Airway responsiveness was measured 24 hours after xylitol exposure in ova-challenged mice and compared to measurements made on naïve mice and ova-challenged mice without any exposure. Mice were anesthetized with Ketamine at 90 mg/kg and Pentobarbital at 50 mg/kg and attached to a small-animal ventilator (Flexivent, SCIREQ). Animals were ventilated at 150 breaths/min. Positive end-expiratory pressure (PEEP) was maintained between 2–3 cmH2O, with the computer setting the tidal volume from the entered weight of each animal. Central airway resistance (R) was measured at baseline and after 10 sec. of nebulized methacholine at doses of 12.5, 25 and 50 mg/ml.

### Safety in normal volunteers

The study was approved by the University of Iowa Institutional Review Board as well as the Food and Drug Administration. Since this is a pilot study and would be the first time xylitol is being used as aerosol, there was no information available on expected complications. Ten subjects aged 18 or greater were studied. Pregnancy or any chronic medical conditions such asthma, atopy, and diabetes were grounds for exclusion. After giving written informed consent subjects underwent a screening spirometry (all subjects demonstrated FEV1 >85% of predicted). Baseline measurements of serum electrolytes, and serum and urine osmolarity were carried out. Baseline oxygen saturation was measured using a pulse oximeter. A brief questionnaire of respiratory symptoms that was developed using a visual analog scale (VAS) was administered at baseline [31,32].

### Human exposures

Subjects received 10 ml of aerosolized saline (generated using a Pari LC Plus nebulizer with Proneb Ultra compressor system, Pari Inc, Monterey, CA) [33]. The particle size of the aerosol was measured using both a 7-stage cascade impactor (Mercer, Inc., Albuquerque, NM) and an Aerosol Monitor (Grimm Technologies, Inc.). The mass median aerodynamic diameter of the aerosol was 1.63 microns with a GSD of 1.71 microns. Mean breathing time for exposures were as follows: Normal saline – 37 min (range 22–49), 1 ml xylitol – 4.2 min (range 2–10), 5 ml xylitol – 22 min (range 15–33), 10 ml xylitol – 36 min (range 30–49).

Thirty minutes after the exposures, subjects completed a follow-up questionnaire, and underwent spirometry and O2 saturation measurement. The procedure was repeated after exposure to 1, 5, and 10 ml of 5% xylitol (Danisco Cultor, USA). Xylitol was prepared by adding 5 gm of crystal sugar to every 100 ml of sterile water (Abbott Laboratories, IL). The solution was sterilized using FDA approved techniques and osmolarity confirmed to be 292 mOsm using a 5500 vapor pressure osmometer (Wescor, Inc., Logan, UT). After completing the exposures, repeat blood and urine tests for electrolytes and osmolarity were carried out. Finally, subjects repeated the symptom questionnaire five days after the first visit, over the telephone. The pre-established criterion for discontinuing study participation was a decline in FEV1 by greater than 20% from baseline.

### Measurement of lung function

Spirometry was performed using a Vmax V6200 Autobox (Sensor Medics Corp., Yorba Linda, CA), according to guidelines published by the American Thoracic Society [34]. The spirometer was calibrated prior to each visit. Spirometry was performed on seated subjects who were using nose clips.

### Respiratory symptom score

The amount of symptoms was assessed at baseline and after each exposure. Subjects scored chest tightness, shortness of breath, cough, headache, chills, muscle soreness, phlegm, nausea, stuffy nose, sneezing, and fatigue on a visual analog scale from 0–10 cm (0 being symptom-free and 10 being extreme amount) [31,32].

### Bronchoscopy and Bronchoalveolar lavage (BAL)

We also examined the effect of aerosolized xylitol on markers of inflammation in the airways. A separate group of subjects underwent bronchoscopy and bronchoalveolar lavage (BAL) according to American Thoracic Society standards at 30 minutes (n = 6), and 3 hours (n = 5) after exposure to 10 ml of aerosolized iso-osmolar xylitol [35]. BAL was performed by instilling two 20-ml aliquots of sterile normal saline into the lingula. The second aspirate was used for cytokine measurements. BAL fluid was filtered through two layers of sterile gauze to remove mucus and centrifuged for 10 minutes at 1500 rpm to separate cells. The cell pellet was washed twice in Hank's Balanced Salt Solution without Ca^++ ^and Mg^++ ^and suspended in complete medium, Roswell Park Memorial Institute (RPMI) tissue culture medium (Gibco/BRL, Gaithersberg, MD). Differential cell counts were determined with cytospin (Shandon, Pittsburgh, Pa) slide preparations by using Wright-Giemsa stain. The cell-free fluid was frozen at -70°C until required for cytokine assay.

Cytokine measurements were performed using enzyme linked immunosorbent assays for IL-6 and LTC-4. IL-6 levels were determined by a Quantikine Human IL-6 ELISA kit (R&D Systems; Minneapolis, MN). The limit of detection of IL-6 is 0.70 pg/ml. LTC-4 (leukotriene) levels were determined by a leukotriene C4 EIA kit (Cayman Chemical; Ann Arbor, MI). The limit of detection of LTC4 is 10 pg/ml. LTC4 of BALs were extracted and concentrated with Cysteinyl-Leukotriene Affinity Sorbent (Cayman Chemical; Ann Arbor, MI).

### Statistical analysis

We studied ten subjects with a gradual increase in exposure dose in the pilot safety study. Differences were analyzed using t-test, Wilcoxon signed rank test, and one way and two-way repeated measures analysis of variance (ANOVA) as indicated. Ninety-five percent confidence intervals were calculated where appropriate. All analyses were performed using SAS version 8.2 (SAS Institute, NC) and at a 5% significance level.

## Results

### Safety in mice

**Figure 1:**
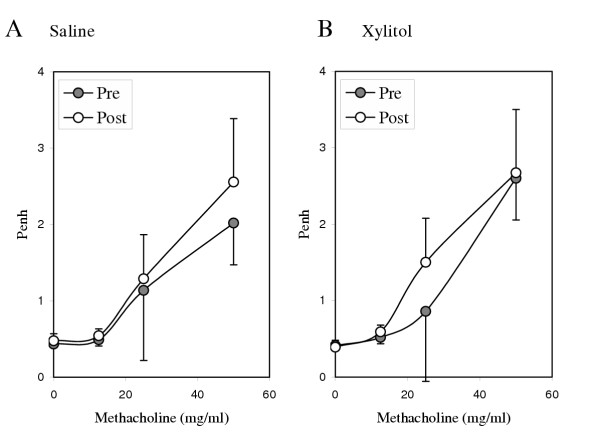
Effect of saline and xylitol exposure on methacholine responsiveness in naïve mice (n = 6/group). Panel A reflects methacholine responsiveness before and after saline exposure. Panel B reflects methacholine responsiveness before and after xylitol exposure. Error bars = SD. P-values of all comparisons are non-significant.

**Figure 2:**
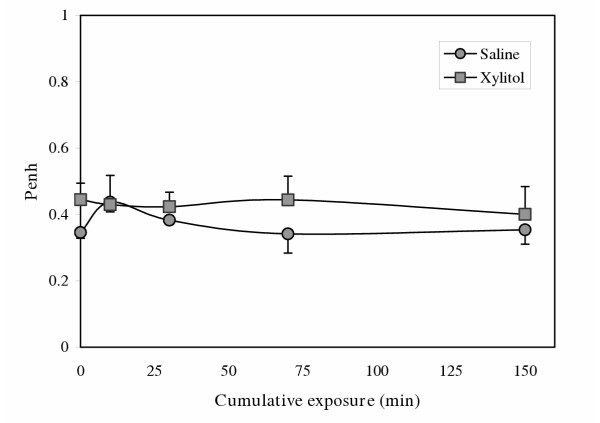
Effect of saline vs. xylitol exposure on Penh of naïve C57BL/6 mice (n = 6). The figure shows mean Penh values for mice exposed to saline (circles) and xylitol (squares). Errors bars = SD. p = 0.21.

**Figure 3:**
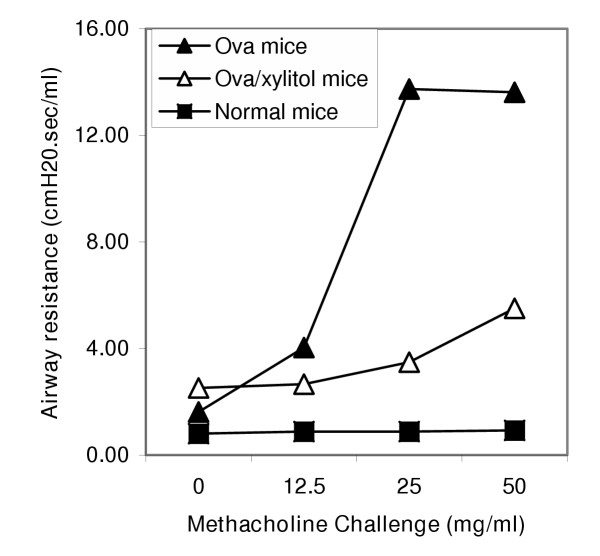
Invasive airway resistance measurement in response to methacholine challenge in naïve and ova-challenged C57BL/6 mice (n = 2/group) using Flexivent system. The figure shows mean airway resistance for naïve mice (squares) ova-challenged mice (triangles).

Mice tolerated the exposures well without any visible distress. The corresponding volume of the 150-minute exposure was approximately 45 ml. Among naïve mice, exposure to xylitol resulted in no significant change in bronchial hyperresponsiveness compared to saline (Figure 1; n = 6/group; p = ns baseline and all concentrations of methacholine). A similar lack of difference between the saline- and xylitol-exposed mice was noted in their tidal volume and respiratory frequencies responses (data not shown). In a separate group of naïve mice that underwent Penh measurements periodically during exposure to saline or xylitol, no significant change was seen in Penh (Figure 2). We carried out similar studies on mice that had been sensitized to, and challenged with ovalbumin, a common murine model of asthma. No significant changes in methacholine responsiveness were observed (data not shown). Figure 3 shows airway resistance measured invasively using the Flexivent system in naïve mice, OVA-sensitized/OVA-challenged mice after saline exposure and OVA-sensitized/OVA-challenged mice after xylitol exposure.

Whole lung lavage showed no significant differences in lavage fluid cell count and differential due to xylitol exposure. Naïve mice exposed to saline or xylitol demonstrated, as expected, a macrophage-predominant response. In contrast, OVA-sensitized/-challenged mice were characterized by airway eosinophilia in both saline- and xylitol-exposed groups (Table 1). In summary, aerosolized xylitol was well tolerated by naïve and hypersensitive mice with no significant effects on the airway physiology or composition of airway inflammatory cells.

**Table 1:** Whole Lung Lavage Cell Count and Differential in Naïve and Ova-challenged Mice

**Experimental Group**	**Total Cell Count (×10^6^) Mean (SD)**	**Differential Count (%)**			
		
		Macrophages	Lymphocytes	Neutrophils	Eosinophils
**Naïve mice-saline**	0.26 (0.8)	99.6	0.17	0.17	0.0
**Naïve mice-xylitol**	0.25 (0.7)	99.0	0.34	0.0	0.66
**Ova-challenged mice – saline exposed**	0.96 (0.1)	20.0	3.6	14.0	62.2
**Ova-challenged mice – xylitol exposed**	0.78 (0.08)	21.3	9.0	9.0	61.0

### Safety in human volunteers

**Figure 4:**
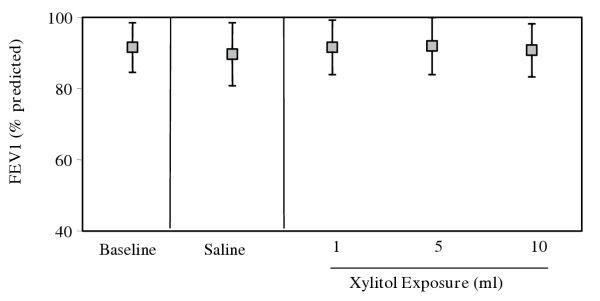
Effect of exposure to nebulized saline and xylitol on spirometry in normal volunteers (n = 10). The figure shows mean FEV1 (% predicted) at baseline, after exposure to saline (10 ml), and xylitol (1, 5, and 10 ml). Errors bars = SD. p = 0.19.

Table 2 shows the baseline characteristics of the ten subjects who underwent graded exposure to aerosolized xylitol as a part of the pilot study. Mean age was 29.1 yrs, and equal numbers of males and females were studied. None of the subjects dropped their FEV1 by ≥ 20%. The mean baseline FEV1 was 92% predicted (SD = 6.9% predicted). There was no significant change in FEV1 % predicted after any exposure in comparison with baseline (Figure 4).

**Table 2:** Baseline Characteristics in Normal Volunteers

**Subject No.**	**Age Years**	**Gender M/F**	**Ethnicity**	**Baseline FEV1 (% predicted)**
1	41	F	Caucasian	92
2	34	M	Caucasian	85
3	48	M	African American	87
4	22	M	Caucasian	106
5	25	M	Asian	95
6	20	F	Asian	85
7	22	M	Caucasian	91
8	20	F	Caucasian	86
9	28	F	Caucasian	100
10	31	F	Caucasian	89
**Mean**	**29**			**92**
**SD**	**9.5**			**6.9**

As shown in Table 3, xylitol exposure did not induce any significant change in electrolytes and osmolarity. No changes in vital signs or oxygen saturation were noted throughout the study. The most common symptom reported was stuffy nose after xylitol exposure, which occurred in five (50%) subjects after the 10 ml dose (Table 4). The mean VAS score among the five subjects for stuffy nose was 3.5 cm. This symptom resolved within minutes after exposure was complete. Other less frequent side effects reported include, cough by two subjects (mean VAS score, 0.5), chest tightness by two subjects (mean VAS score, 1.0), and phlegm production by three subjects (mean VAS score, 1.5). All of these symptoms had resolved by day five of telephone follow-up. One subject noted hiccups half way through the final xylitol exposure, which resolved soon after the exposure was complete.

**Table 3:** Laboratory Results pre and post Xylitol Exposure (n = 10)

**Serum test**	**Baseline Mean ± (SD)**	**After 10 ml xylitol Mean ± (SD)**	**p value**
**Glucose, mg/dL**	89 (3.8)	89 (9.1)	0.98
**Osmolarity, mosm/k**	292 (5.2)	292 (3.9)	0.98
**Sodium, mEq/L**	141 (1.4)	141 (2.6)	0.75
**Bicarbonate, mEq/L**	25 (1.2)	24 (1.9)	0.41
**Anion gap, mEq/L**	13 (1.2)	13 (1.2)	0.69

**Table 4:** Adverse Events Score (centimeters, mean ± SD) using Visual Analog Scale (1–10)*

**Symptom**	**Baseline VAS score**	**Change Post-saline**	**Change Post-10 ml xylitol**	**Change on day 5 follow-up**
**Chest tightness**	0	0	0.2 ± 0.4	0
**Shortness of breath**	0	0	0	0
**Cough**	0.25 ± 0.8	0.05 ± 0.15	0	0
**Headache**	0	0	0.2 ± 0.6	0
**Chills**	0	0	0	0
**Muscle soreness**	0.2 ± 0.6	0	0	-0.2 ± 0.6
**Phlegm**	0.2 ± 0.6	0	0.25 ± 0.4	0
**Nausea**	0	0	0	0
**Stuffy/Runny Nose**	0	0	0.65 ± 0.9†	0
**Sneezing**	0	0	0	0
**Fatigue**	0.1 ± 0.3	0	-0.1 ± 0.3	0

*P-values of all changes from baseline are >0.05 except for stuffy nose after xylitol expsoure.

† P-value = 0.03.

An additional 11 subjects underwent bronchoscopy and bronchoalveolar lavage following xylitol inhalation. The mean cell count in the BAL fluid at 20 minutes (n = 6) and 3 hours (n = 5) after xylitol exposure was 1.2 ± 0.07 million cells/ml and 2.94 ± 1.48 million cells/ml respectively. All cell preparations had between 95–100% alveolar macrophages. BAL IL-6 and LTC-4 levels after xylitol exposure were below 0.70 pg/ml and 10 pg/ml respectively at all time points.

## Discussion

Lower respiratory tract colonization is an important step in the pathogenesis of pulmonary manifestations of chronic diseases such as CF and dyskinetic cilia syndrome and certain acute clinical entities such as ventilator-associated pneumonia. There is a continuing need for simple, cost-effective, and safe intervention to decrease colonization of lower airways. Studies have shown that lowering the salt concentration of airway surface liquid can enhance innate immunity by increasing the potency of the natural antimicrobial peptides. In addition to increasing the activity of individual ASL factors, lowering the NaCl concentration also independently enhances synergistic interactions [36]. Thus, lowering the salt concentration could improve the antimicrobial activity of the ASL in two ways: increasing the individual action of the factors, and augmenting synergism between them. This double effect could amplify the impact of relatively modest reductions in salt concentrations. The mechanism of this low salt concentration augmentation of killing remains unclear. The most popular hypothesis is that in low salt concentrations, charged particles become less shielded, increasing the interaction between the cationic proteins and the negatively charged bacteria [6,7,37,38]. Irrespective of the mechanism, this effect suggests a therapeutic strategy: lowering ASL salt concentrations should enhance bacterial killing.

Xylitol, when applied to airways as an iso-osmolar agent, can potentially lower airway salt concentration and therefore lower bacterial colonization in chronic infections. In addition to having low transepithelial permeability, it has the added advantage of being poorly metabolized by bacteria. In recent years, many osmotic agents have been aerosolized into human airways for mucus clearance. However, there are reports of bronchospasm associated with their use. This is the first study to our knowledge to use xylitol in an aerosolized form.

The main adverse effect reported from oral xylitol use was diarrhea when the dose exceeded 40–50 gm/day [39]. Intravenous xylitol has also been used as parenteral nutrition in the post-operative period for many decades. There have been no major changes in serum electrolytes with xylitol infusion [40]. Parenteral xylitol can cause minimal hyperuricemia, but without any pathophysiological consequences [41]. Though tolerated well in modest doses, large doses of xylitol administered intravenously have been reported to cause renocerebral oxalosis, with renal failure [42-45]. Before xylitol use in humans for prevention or reduction of airway colonization can be attempted, animal studies on safety as well as studies on healthy volunteers are required.

We made calculations of the amount of xylitol to be delivered to the airway surface of an adult. Mercer, *et al*. [46] measured a total surface area from trachea to bronchioles of 2,471 cm^2^. The depth of ASL may vary from the trachea to the small bronchioles; if an average depth of 10 μm is estimated, the total ASL volume would be ~2.5 mL. Thus, if we assume a uniform aerosol distribution, administration of a total volume of 2.5 mL of 300 mM xylitol to the airways would be expected to lower the salt concentration in half simply by a dilutional effect. If the mean ASL depth were 20 μm, then 5 mL of delivered solution would be required. Because the solution is iso-osmotic, immediate, major osmotic shifts of water across the epithelium should not occur, which leads to dilution of the salt concentration. Moreover, with time, the volume and salt concentration may decrease due to Na^+^-dependent salt absorption, the osmotic effects of which are counterbalanced by xylitol in the ASL [13].

Our preliminary calculations for dosing for mice experiments were derived as follows; Mercer, *et al*. [46] also estimated the total airway surface area in rats, which was 27.2 cm [3]. Assuming an average depth of 10 μm, the total ASL volume would be ~27 μl. For a mouse, given an average weight of 25 gm, which is 1/12th of weight of a rat, the ASL volume is approximately 2.25 μ l. For a 50% dilution we have to deliver 2.25 μl of xylitol solution. Mice have an approximate 10% lung retention rate for the particle size we generated [47], which will require us to aerosolize 22.5 μl of xylitol. However, we do not have data on the airborne concentration of xylitol to which the mice were exposed. For the generation and exposure system employed, a reasonable approximation is that 5% of the solution nebulized into the mixing chamber was available for inhalation in the exposure chamber. Thus, we would need to deliver approximately 450 μl of xylitol solution to provide the desired 50% dilution of ASL. We exposed both normal and hypersensitive mice to a cumulative volume of 84 ml of iso-osmotic xylitol, which is at least a 2-log increase (187×) from the proposed dose. There was no significant change in airway resistance nor in bronchial hyperresponsiveness after xylitol exposure in naïve or hypersensitive mice.

This study shows that aerosolization of iso-osmotic xylitol is likely to be safe and well tolerated by human volunteers. There was no change in spirometry, laboratory test results as well as BAL cytokine levels after xylitol exposure. Earlier studies have reported bronchial hyperresponsiveness with aerosolization of hypotonic and hypertonic solutions. Thus, aerosolization of iso-osmotic xylitol could be tested for prevention and treatment of airway colonization.

There are several potential limitations with this study. The validity of body plethysmography as a measure of respiratory physiology in mice has been recently questioned [48,49]. However, several studies have shown good correlation between airway inflammation and changes in Penh [50-52]. Since the human study is a true pilot study, we did not have preliminary data on adverse events for the aerosolized route to base our sample size calculation; given its relatively small size, we do not have the power to detect rare complications. Our human study was unblinded due to the sweet taste of xylitol, which all the subjects experienced. However, our main outcome, FEV1 is unlikely to be biased by knowledge of the exposure. Finally, this was a brief exposure study. Inhalational toxicology studies of safety of long-term exposure to animals looking at histopathology and laboratory data in addition to pulmonary function testing are required before clinical use can be instituted.

## Conclusions

In summary, our data indicate that iso-osmotic xylitol can be safely delivered by aerosol to normal volunteers. Studies of safety with long-term exposure to animals are required before human use can be attempted. This could lead to exciting interventions to enhance the innate immunity of airway epithelia.

## Abbreviations

ANOVA Analysis of Variance

ASL Airway Surface Liquid

CF Cystic Fibrosis

FEV1 Forced Expiratory Volume in 1 second

GSD Geometric Standard Deviation

Penh Enhanced Pause

VAS Visual Analog Scale

BAL Bronchoalveolar Lavage
